# Why we need a single independent international hypertension clinical practice guideline

**DOI:** 10.1038/s41440-021-00666-6

**Published:** 2021-05-12

**Authors:** Mohamed Ben-Eltriki, Alan Cassels, Juan Erviti, James M. Wright

**Affiliations:** 1 Cochrane Hypertension Review Group, University of British Columbia, Vancouver, BC Canada; 2 Therapeutics Initiative, University of British Columbia, Vancouver, BC Canada; 3 Department of Anesthesiology, Pharmacology and Therapeutics, Faculty of Medicine, University of British Columbia, Vancouver, BC Canada; 4 Department of Medicine, Faculty of Medicine, University of British Columbia, Vancouver, BC Canada

Clinical practice guidelines are assumed to be based on the best available evidence using the principles of evidence-based medicine. Because hypertension is a global problem and the global body of high-quality evidence is available to all, we question the justification for individual countries developing their own guidelines. Unfortunately, many countries have indeed developed and published country-specific hypertension guidelines [1]. We use this fact as an opportunity to explore whether countries use a similar evidence-based process and avoid bias due to conflicts of interest.

The cornerstone of the evidence-based process is the systematic review (SR), a concise summary of the best available evidence that addresses a sharply defined clinical question and attempts to answer it using explicit and rigorous methods to identify, critically appraise, and synthesize all relevant studies. Hypertension guideline developers could start from scratch and conduct SRs to answer the many clinical questions that arise during the diagnosis, management, and drug treatment of hypertension. However, doing so would require a large group working over a long period of time. A much more efficient and pragmatic approach is to carefully select the best available high-quality evidence from hundreds of published SRs.

Currently, a large number of SRs on hypertension have been edited by the Cochrane Hypertension Review Group and published in the Cochrane Library. The main objective of the Cochrane Hypertension Group is to publish reviews answering the most important questions related to hypertension. These reviews have the advantage of following gold-standard Cochrane methods, which are transparent and rigorous. Furthermore, Cochrane reviews are required to be updated regularly.

The Cochrane Hypertension Group has recently been challenged with the question of how a nonexpert could quickly and easily assess whether a hypertension guideline is based on the best available evidence and is free from bias due to potential conflicts of interest. We came up with a simple two-step exercise. First, check the reference list and report how many SRs are cited. Second, document the funding sources of the development process and the conflicts of interest among the authors.

To test this exercise, we compared the Canadian [2] and Malaysian [3] hypertension guidelines. We chose the Hypertension Canada guideline because that is where the editorial headquarters of the Cochrane Hypertension Group is situated. As a comparison, we chose the Malaysian guideline because Malaysia differs from Canada geographically, racially, and culturally. Table 1 compares the use of SRs in the two most recent Canadian and Malaysian guidelines. A summary of the potential conflicts of interest in the two most recent Canadian and Malaysian guidelines is shown in Table 2.

**Table 1:** Use of systematic reviews in two hypertension guidelines

Items	2020 Hypertension Canada	2018 Malaysian Guideline
Total references cited	111	506
Cochrane hypertension SR cited	0	12
Other Cochrane SR cited	0	11
Total non-Cochrane SR cited	10	89
Total SR cited	10	112

*SR* systematic review

**Table 2:** Potential conflicts of interest in two hypertension guidelines

Items	2020 Hypertension Canada	2018 Malaysian Guideline
Funding of guideline development	Hypertension Canada	Servier Malaysia
Number of authors	82	26
No declared conflicts	49	26
Declared conflicts	33	
Research grants/support	12	
Travel support	3	
Speaker fees	12	
Honoraria	14	
Consultancy	11	
Director/president	2	
Advisory Boards	10	
Stockholder	1	
Employed	1	
Member	1	
Intellectual Property Rights	1	

This simple exercise demonstrates a markedly different process for producing hypertension guidelines in two countries. The Malaysian guideline made much more use of SRs (see Table 1). If we accept that SRs are the foundation of the evidence-based process, it is likely that the Malaysian guideline is more evidence-based than the Canadian guideline. We were surprised that the Canadian guideline cited few SRs and no Cochrane reviews given that the evidence-based medicine movement originated in Canada at McMaster University in 1990 [4].

In terms of preventing bias, both guidelines state that they have attempted to prevent conflicts of interest in the guideline development process. The Malaysian guideline admits that it received funding from a drug company, Servier, but states that the company had no role in or influence on the recommendations. It is not easy to determine how Hypertension Canada is funded, but the funding most likely comes from drug companies that make antihypertensive drugs. In both cases, how these potential conflicts of interest were managed was not transparent. The Malaysian guideline development group managed author conflicts of interest by accepting only individuals with no competing financial or professional interests as members of its guideline development committee. The Canadian guideline allowed people with potential conflicts as long as the conflicts were declared and recorded. It also stated, “The members of Canadian guideline recused themselves from voting on specific guidelines”; however, we have no information as to how that recusal was achieved.

We assume that the objectives in both countries were to optimize the diagnosis, management, and treatment of high blood pressure to achieve the best possible outcomes for their populations. Guidelines, regardless of where they are developed, must follow a consistent, rigorous and transparent process. To accept guideline recommendations, the profession and the public deserve a clear explanation of the reasoning linking the evidence to the recommendations. High-quality and comprehensive SRs represent the best available evidence. The reality is that elevated blood pressure (hypertension) is the same condition worldwide and is a risk factor for adverse cardiovascular events. The evidence base is available and the same everywhere in the world. Therefore, the optimal diagnosis and management of hypertension should also be the same around the world.

Our simple exercise demonstrates that the process of producing the same type of guideline in two countries is markedly disparate, exemplifying why individual countries creating their own guidelines is problematic. We have deliberately not tried to compare or critique the recommendations in the two guidelines. However, as one would expect, there are noticeable differences in recommendations concerning treatment targets and choice of first-line antihypertensive drugs. One can imagine the confusion this creates for students, clinicians, and the public worldwide.

We believe that an independent body with no conflicts of interest should be established to develop evidence-based hypertension guidelines for the world. The advantages of this approach in terms of providing a unified consistent message and saving time and effort are obvious. The advantage in terms of minimizing bias is even greater. Such an international cooperative effort would do well to consider the contributions and expertise of the Cochrane Collaboration. The Cochrane Hypertension Group and other groups are continuously attempting to ensure that the most important clinical questions regarding hypertension are answered and that the reviews are kept up-to-date. Cochrane is aware that gaps in the knowledge base exist and is open to working with authors to help fill these gaps.

An international hypertension guideline would still need the involvement of groups in individual countries to promote it and translate it into the appropriate languages. Any country that felt it had unique local issues, such as evidence for a different approach based on race, that were unaddressed in the international guideline could produce an addendum for its own local use [5].

In conclusion, hypertension guidelines should be evidence-based and follow the principles of evidence-based medicine independent of where they are developed. We document a surprisingly different use of SRs and a different approach to reducing conflicts of interest in the Canadian and Malaysian hypertension guidelines. We realize that this topic will be controversial, but as such, it will be engaging and interesting for the scientific community. We use this example to make a strong appeal for a single independent international hypertension guideline for use worldwide.
